# Chromosome Segregation in the Oocyte: What Goes Wrong during Aging

**DOI:** 10.3390/ijms23052880

**Published:** 2022-03-07

**Authors:** Marta Wasielak-Politowska, Paweł Kordowitzki

**Affiliations:** 1 Center of Gynecology, Endocrinology and Reproductive Medicine–Artemida, Jagiellonska Street 78, 10-357 Olsztyn, Poland; m.wasielak.politowska@gmail.com; 2 Institute of Animal Reproduction and Food Research of Polish Academy of Sciences, Tumiwa Street 10, 10-243 Olsztyn, Poland; 3 Department of Basic and Preclinical Sciences, Institute for Veterinary Medicine, Nicolaus Copernicus University, Gagarina Street 1, 87-100 Torun, Poland

**Keywords:** spindle formation, spindle assembly, euploidy, aneuploidy, oocytes, maternal aging, chromosome segregation

## Abstract

Human female fertility and reproductive lifespan decrease significantly with age, resulting in an extended post-reproductive period. The central dogma in human female reproduction contains two important aspects. One is the pool of oocytes in the human ovary (the ovarian reserve; approximately 10^6^ at birth), which diminishes throughout life until menopause around the age of 50 (approximately 10^3^ oocytes) in women. The second is the quality of oocytes, including the correctness of meiotic divisions, among other factors. Notably, the increased rate of sub- and infertility, aneuploidy, miscarriages, and birth defects are associated with advanced maternal age, especially in women above 35 years of age. This postponement is also relevant for human evolution; decades ago, the female aging-related fertility drop was not as important as it is today because women were having their children at a younger age. Spindle assembly is crucial for chromosome segregation during each cell division and oocyte maturation, making it an important event for euploidy. Consequently, aberrations in this segregation process, especially during the first meiotic division in human eggs, can lead to implantation failure or spontaneous abortion. Today, human reproductive medicine is also facing a high prevalence of aneuploidy, even in young females. However, the shift in the reproductive phase of humans and the strong increase in errors make the problem much more dramatic at later stages of the female reproductive phase. Aneuploidy in human eggs could be the result of the non-disjunction of entire chromosomes or sister chromatids during oocyte meiosis, but partial or segmental aneuploidies are also relevant. In this review, we intend to describe the relevance of the spindle apparatus during oocyte maturation for proper chromosome segregation in the context of maternal aging and the female reproductive lifespan.

## 1. Introduction

Following great improvement in education and lifestyle, maternal age has significantly increased in developed countries compared with the last few decades, and in parallel, the age at which women have their first child has also increased [1]. One of the major threats of advanced maternal age is the decline of fertility, as the mammalian ovarian microenvironment experiences profound effects of aging very early in life [2,3]. The inverse relationship between age and fertility in women is generally believed to be due to a reduction of the ovarian follicle reservoir and the chronic exposure of the ovarian microenvironment to different aging-related stimuli. However, the influence of oocyte quality on the reproductive capacity of aged females remains unclear [4]. Interestingly, the pregnancy rate was restored to normal young females when oocytes from young females were fertilized in vitro and transferred to aged-matched recipients [5,6]. This reinforces the concept that oocyte quality is the key to fertility in advanced-age women. 

**Figure 1:**
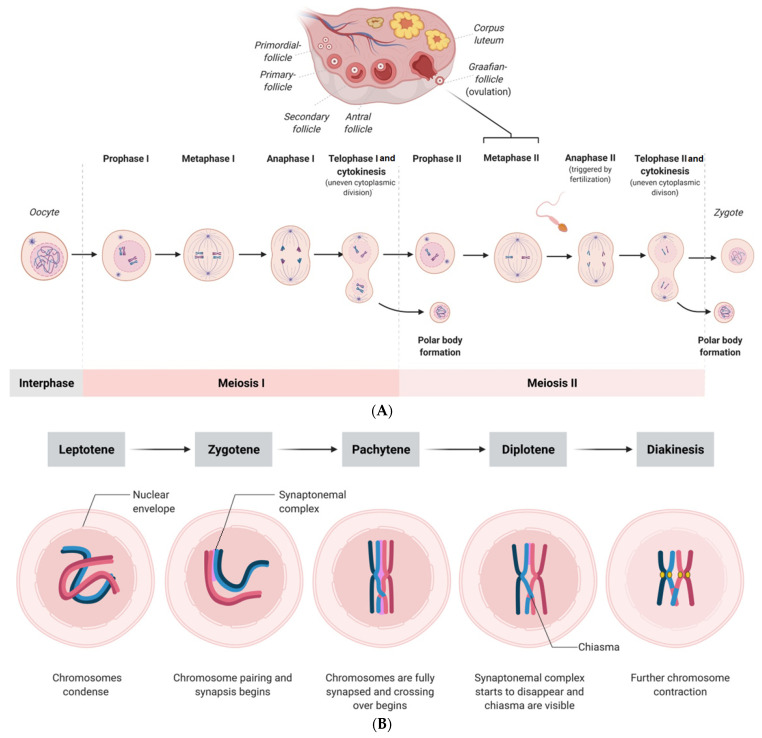
(**A**) Scheme showing the processes of meiosis I and II of the human oocyte and the zygote formation after fertilization. (**B**) Scheme showing the physiological stages of prophase I (first meiotic division) in oocytes, which consists of the leptotene, zygotene, pachytene, diplotene, and diakinesis stages. (For better visualization, the spindles are shown larger than their physiological size compared with the size of the oocyte).

Multiple potential mechanisms have been suggested to be responsible for the age-associated decline in oocyte quality, such as mitochondrial dysfunction, epigenetic alterations, DNA damage, and chronic exposure to oxidative stress, which are not discussed in this review. Another crucial factor for the aging-related decline of oocytes is the meiotic spindle apparatus, which has piqued researchers’ interest. During mammalian oocyte maturation, proper spindle assembly ensures even distribution and segregation of chromosomes during meiosis [7]. Herewith, there are fundamental differences in meiotic divisions; in meiosis I, homologous chromosomes are segregated, while in meiosis II, sister chromatids are segregated (Figure 1) [7]. Interestingly, aging also affects chromatin remodeling, and there are known mitotic errors that might occur during the cell cleavage of the early embryo. It happens that embryos contain both euploid and aneuploid blastomeres, and notably, mosaicism is also detected in embryos with correct morphology, which is described in more detail in Section 4. Prior to the selection for further development, all human oocytes are arrested in prophase of the first meiotic division. The stages leptotene, zygotene, pachytene, diplotene, and diakinesis are depicted in Figure 1. Once a follicle bearing the oocyte is selected for ovulation, adequate segregation of chromosomes (euploidy) during mammalian meiosis is crucial for physiological cleavage and early embryonic development after fertilization [8]. Otherwise, aberrant segregation of chromosomes (i.e., aneuploidy) may result in miscarriage or birth defects. Over the past few years, various mechanisms involved in maternal aging-associated aneuploidy have been reported [9]. A better understanding of novel interactions and pathways involved in mammalian oocyte spindle assembly, especially in oocytes of women with advanced reproductive age, can lead to new therapeutic strategies. Therefore, preimplantation genetic testing to detect chromosome segregation failures would significantly improve the selection of the blastocyst prior to its transfer, although there are ethical issues that cannot be neglected.

In this review, the significance of proper meiotic spindle assembly of the mammalian oocyte in advanced maternal age and its importance for chromosome segregation is presented. Additionally, this review covers specific key pathways, checkpoints of meiotic spindle formation, and the causes of spindle abnormalities. Finally, we describe the aspects related to euploidy relevant for in vitro fertilization (IVF) clinics.

## 2. Impact of Proper Spindle Formation on Euploidy

**Figure 2:**
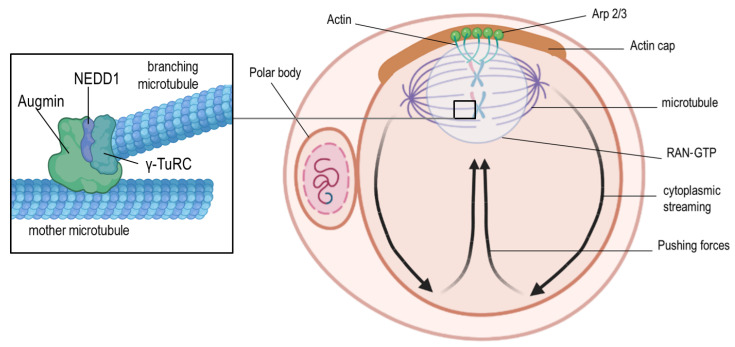
Positioning of the meiosis II spindle. At the end of meiosis I, the spindle and the cortical cap interact to generate mechanical forces that lead to the extrusion of the first polar body. The cortical actin-rich domain (actin cap) is formed through the chromosome-associated RAN-GTP-dependent signaling after spindle migration to the cortex during meiosis I. After the extrusion of the first polar body, meiosis II is initiated, which requires the assembly and active maintenance of the spindle near the actin cap through RAN-GTP-dependent chromatin signaling. As a consequence, the Arp2/3 complex is activated to induce actin filament nucleation and support retrograde actin flow along the lateral cortex and back toward the oocyte center. This cytoplasmic streaming in the direction of the actin cap pushes the spindle toward the cap domain. Microtubule nucleation is depicted in the left zoom-in box, showing the Augmin complex, which recruits γ-TuRCs at the surface of the “mother microtubule” to initiate nucleation of “branching microtubules”. The Augmin-dependent recruitment takes place in the presence of NEDD1 (for better visualization, the pushing forces are depicted with black arrows, and spindles are shown larger than their physiological size compared to the size of the oocyte).

Proper spindle formation in oocytes ensures error-free meiosis. It involves a sequence of events, such as microtubule nucleation, organization in a bipolar fashion, and chromosome alignment at the equator of the spindle, to enable correct segregation (Figure 1). Similar to the mitotic spindle, the meiotic spindle is bipolar; however, the spindles differ in the microtubule organization process [10]. In human oocytes, the so-called chromatin-based RAN-GTP gradient is essential for microtubule nucleation [11,12]. The RAN-GTP gradient surrounding the chromosomes (Figure 2) activates local spindle assembly factors responsible for microtubule polymerization [11,13,14,15]. Blocking its function results in spindle instability and improper kinetochore–microtubule connections, followed by chromosome segregation defects [11].

Different models of chromosome assembly at the meiotic spindle equator have been proposed in the past. During mitosis, the “search and capture” model relies on the capture and stabilization of centrosome-nucleated microtubules by the kinetochores of both sister chromatids of each chromosome [16]. However, in oocytes, “self-assembly” of chromosomes takes place. Accordingly, kinesin-like proteins localized along chromosome arms generate polar ejection forces, causing chromosome alignment at the metaphase plate [17]. The premature segregation of the chromosomes is blocked by spindle assembly checkpoint (SAC) proteins, while all kinetochores are not. The spindle assembly checkpoint mechanism was thought to be specific to mitosis; however, its function in preventing anaphase onset during meiosis was later confirmed [18,19]. The presence of several SAC proteins (MAD1, MAD2, BUB1, BUB1R, BUB3, and MPS1) was confirmed in murine oocytes [20]. Maintaining the accuracy of chromosome alignment at the metaphase plate during meiotic divisions plays a significant role in aneuploidy prevention.

Asymmetric cytokinesis is an attribute of both meiotic divisions in oocytes and results in the formation of the large oocyte and the small polar bodies. It is well-established that spindle relocation from the center to the cortex of the oocyte is guided by the cytoplasmic actin microfilaments [21,22]. Deficiencies in Arp2/3 (Figure 2), known for their role in the actin nucleation process, result in the disturbance of spindle movement to the oocyte periphery and the lack of the first polar body [23,24]. The polar body is possibly generated through the contractions of actin and myosin II, forming a ring between the spindle poles [25]. When the first polar body finally extrudes into the perivitelline space, the second meiotic spindle forms parallel to the oolemma. Finally, sperm penetration induces its rotation to a vertical position, meiosis II completion, and second polar body formation. It was found that in murine oocytes, cytochalasin B treatment inhibited spindle rotation and cytokinesis. Consequently, an extra pronucleus was formed, highlighting the role of actin filaments in this process [26]. A recent study postulated that the organization of the Arp2/3 complex (Figure 1) and the myosin II filaments on top of the anaphase II spindle is asymmetric and regulates the acting forces of spindle rotation [27]. This study also proposed the existence of two feedback pathways, namely between the RAN-GTP gradient and the Arp2/3 complex, and the suppressing crosstalk between the Arp2/3 actin network and the myosin II network (Figure 2) [27]. This hypothesis has been supported by findings of other studies [28,29,30,31,32], but it is still not fully understood how the RAN-GTP mechanism can change the association or the activity state of factors required for cytoskeletal rearrangements. Further research is necessary to elucidate the multifaceted relationships between Arp2/3 and the actin network [27].

Interestingly, microtubule organizing centers (MTOCs) in oocytes usually do not possess centrioles; however, they contain pericentriolar matrix (PCM) proteins, which are typical for mitotic centrosomes, such as pericentrin, γ-tubulin, NEDD1, and nuclear mitotic apparatus protein (NuMA) [33]. In mice, the lack of pericentrin or other PCM proteins disturbed the meiotic spindle assembly and caused improper alignment of the chromosomes [34]. The RAN-GTP pathway in the murine species is involved in the later stages of microtubule formation, whereas the initial phase is MTOC-dependent [35]. These MTOCs are arranged in large clusters in murine eggs during the germinal vesicle stage; when meiotic maturation starts, they are scattered into small foci and distributed along the nuclear envelope to reach the chromosomes after nuclear envelope breakdown [36,37]. Notably, the lack of functional MTOCs in murine oocytes leads to an error in spindle assembly, improper attachment of microtubules to the chromosomes, and, consequently, aneuploidy [38,39]. Two other pathways are involved in microtubule nucleation and spindle assembly in oocytes, namely the Augmin-dependent and chromosomal passenger complex (CPC)-dependent pathways. The latter-mentioned complex is composed of the inner centrosome protein (INCENP), borealin, survivin, and Aurora B kinase, and its recruitment takes place at the centromeres. Previous studies have suggested that the kinase subunit of CPC might be responsible for the phosphorylation and inactivation of local microtubule-depolymerizing proteins, such as MCAK [40] and Op18/Stathmin [41]. This facilitates the stabilization of microtubules and leads to spindle formation [42]. However, microtubule depolymerization within the spindle is prevented by an Aurora B diffusion gradient present in the entire spindle, although CPC concentrates at each centromere [43,44]. Moreover, Augmin influences the localization of the γ-tubulin ring complex (γ-TuRC), a potent microtubule nucleator [45]. It has been shown that, in the case of the Augmin pathway, disturbances such as Augmin depletion or inhibition of the Augmin–γ-TuRC interplay (Figure 2) causes polymerization of the microtubules and leads to MTOC clustering errors. These errors of the MTOC/centrosome clustering due to the lack of Augmin might be the consequence of a microtubule nucleation failure within the spindle or errors in other pathways that are dependent on Augmin, such as the microtubule bundling or crosslinking pathways [45].

In summary, the RAN-GTP gradient is crucial for microtubule nucleation, especially in human eggs, and the importance of MTOCs and their interplay with other proteins cannot be neglected. How advanced maternal age influences these processes will be discussed in the following section.

## 3. Effect of Advanced Maternal Age on Chromosomal Segregation Accuracy

Perhaps the most well-documented effect of the aging-related phenotype in the oocyte is chromosome segregation failure, in other words, the increasing prevalence of aneuploidy. This hallmark of aging in oocytes frequently occurs during the first meiotic division. Aneuploidy is known to be the cause of miscarriage and mental retardation [46,47] and can result from different defects in segregation, namely the reverse segregation or premature separation of sister chromatids or meiosis I nondisjunction. Chromosomal analysis of human and mouse oocytes has revealed that the chance of aneuploidy increases by more than 50% in advanced aged [48,49]. Interestingly, euploid oocytes selected from older women possessed similar implantation potential to those of their younger counterparts in IVF [50], suggesting that aneuploidy is the predominant cause of female fertility reduction. Although the exact mechanism of the age-induced aneuploidy rate increase is yet to be fully understood, it has been suggested that chromosome segregation error related to SAC defects and loss of cohesion are the major contributing factors in aneuploidy. The SAC is triggered by unattached kinetochores. In a recent study on mice oocytes, it was revealed that AURKB appears to play a role in the maintenance of the female reproductive lifespan, most likely because of its defensive property against the excessive accumulation of reactive oxygen species in the egg [51]. However, this is only one possible factor among others. Therefore, further investigations on the abundance of AURKB with respect to the advancing age of the egg could shed light on its ability to be used as an adequate biomarker for oocyte developmental competence in women with aging-related decreases in fertility [51].

As mentioned in the previous sections, the SAC is a signaling system composed of different proteins, such as monopolar spindle 1 (MPS1) [52], members of the budding uninhibited by benzimidazole (BUB) protein family, and members of the mitotic arrest-deficient (MAD) protein family. MAD proteins have a suppressive effect on the E3 ubiquitin ligase anaphase-promoting complex/cyclosome (APC/C), which prevents the onset of anaphase prior to complete and correct kinetochore–microtubule attachment [53,54,55]. In previous mouse models in which specific SAC components, such as Mad2 [56,57,58], Bub1 [59,60], and BubR1 [56,61], were expressed at low levels, the oocytes showed an increase in chromosome segregation errors and spindle defects. This emphasizes the impact of SAC on aneuploidy in oocytes. Differences in the expression levels of SAC proteins have been reported when murine oocytes of young donors were compared with those of their advanced-age counterparts [62]. Oocytes with a SAC deficiency failed to retard meiosis I [57,58,63]. On one hand, researchers have suggested that a defective SAC may not be the primary cause of age-related oocyte aneuploidy [64,65]. On the other hand, there is strong evidence that an impaired ability to arrest the anaphase onset, along with a much lower rate of microtubule error correction in aged oocytes, could represent a more relevant aging phenotype [66]. This supports the hypothesis that a progressive dysfunction of an error-prone SAC may be the leading cause of age-related oocyte aneuploidy.

Along with the relevance of the SAC proteins, age-associated chromosomal segregation failures might be due to weakened cohesion between sister chromatids during meiosis I [67,68,69,70,71,72]. Following chromosome duplication, sister chromatids within each pair of homologous chromosomes are held together by the cohesion complex, which contains four main components: Smc1b, Smc3 [71,73], Rec8 [74], and Stag3 [75]. It has been shown that Rec8 needs to be cleaved by separase in murine oocytes [76]. One of the Shugoshin proteins, Sgo2, was shown to be mandatory for the protection of centromeric cohesion and correct chromosome segregation during meiosis I [77]. The knockdown of Sgo2 resulted in a lack of centromeric cohesion protection during oocyte meiosis I [78]. Aging has been associated with a reduction in cohesion, which most likely represents one of the main causes of age-associated aneuploidy in mammalian oocytes [79]. However, Smc1b deficiency was found to result in cohesion loss, reduction of chiasmata, increased univalent chromosomes, and a significantly higher aneuploidy rate [69,71]. Depletion of another cohesin subunit, Rec8, at different stages of oogenesis resulted in the loss of chiasmata, which could not be rescued upon postnatal Rec8 overexpression [80]. Interestingly, the inhibition of sirtuin expression by rapamycin led to a disruption in Cdk1 regulation, resulting in impaired entry into meiosis I and the establishment of meiosis II arrest [81]. The discovery of BubR1, a SAC component, as a novel de-acetylation target of SIRT2 in mice raised interest in the role of sirtuins in oocyte biology [82,83]. The reduction of SIRT2 in vitro and in vivo led to a decreased level of the BubR1 protein [82]. Moreover, maintaining the expression of NAD+ in aged murine oocytes comparable to the level in young oocytes prevented defective spindle integrity and improved the live birth rate [83].

In summary, aneuploidy in human eggs is caused by the nondisjunction of entire chromosomes or sister chromatids during oocyte meiosis, but partial or segmental aneuploidies were also described [84], resulting from different defects in segregation, namely reverse segregation, premature separation of sister chromatids, or meiosis I nondisjunction. The clinical relevance of aneuploidy, especially for female patients in human infertility clinics, is described in more detail in the following section.

## 4. Spindle Formation Disturbances and the Clinical Relevance of Aneuploidy 

The process of chromosome segregation is extremely sensitive, even to minor changes in the timing or biochemistry of spindle formation. Confocal microscopy studies have shown that human oocytes from women over 40 years of age who reproduce naturally have a high incidence of meiotic spindle aberrations, such as altered tubulin localization and the displacement of the chromosome from the metaphase plate of meiosis II [85]. Analogous observations were made in oocytes subjected to in vitro aging. The experiments demonstrated that their prolonged culture for one or two days resulted in shorter, multipolar, or disorganized spindles. As a result, chromosomes at the metaphase plate were not properly arranged but scattered within the degenerating spindle [86,87,88]. In experiments with aged human oocytes in vitro, the integrity of spindle stability regulators, such as microtubule-associated proteins, was also impaired [89]. Moreover, the expression of other SAC complexes (Ran, Tpx2, and Dcor1) and kinetochore-associated proteins (MAD2 and BUB1) was also affected by aging in human oocytes [90,91,92]. 

During the IVF procedure, the meiotic spindle of oocytes is very susceptible to disruption due to temperature imbalances. This issue has been addressed in many studies investigating the effects of temperature alterations on ultrastructural and molecular characteristics of human oocytes [93,94,95,96,97]. It is known that even brief exposure of the oocytes to a temperature slightly below 37 °C causes depolymerization of tubulin, disruption of the spindle structure, and damage to chromosomes [96]. Changes in the spindle apparatus, such as the reduction of its size and the disorganization of microtubules, were observed in oocytes kept at room temperature for 30 min compared with control oocytes constantly kept at 37 °C [93]. Similarly, increasing the temperature to 40 °C resulted in spindle degradation. When the temperature was lowered back to 37 °C, the repolymerization of microtubules was observed; however, the spindles were not fully restored [98]. Therefore, maintaining an adequate temperature throughout the in vitro process of oocytes is crucial to maintain normal spindle morphology and ensure proper chromosome segregation during meiosis II. Considering the high sensitivity of the spindle to low temperatures, it seems reasonable that the cryopreservation of oocytes may result in lower fertilization rates and reduce subsequent embryonic development [99]. However, spindle recovery after freezing and thawing differ depending on the cryopreservation method. In the case of slow freezing, up to half of the human and bovine oocytes matured in vitro had abnormal spindle and chromosome morphology [100,101,102]. By contrast, better results were obtained when vitrification was applied to mature oocytes derived from super-ovulated donors [103,104]. In addition, clinical studies have shown that vitrification of oocytes does not increase the aneuploidy of the resulting embryos [105]. Nevertheless, some authors noted an increased frequency of chromosome alignment disorders and aneuploidy after vitrification [106,107].

Findings related to the incidence of aneuploidy in oocytes that were matured in vitro compared with those matured in vivo are contradictory. On one hand, a study by Cooper et al. [108] provided evidence that there was no difference in the aneuploidy rate of murine oocytes that were obtained in vivo or generated upon IVM. On the other hand, further studies have reported that the IVM procedure impacted the meiotic spindle size and shape; additionally, the risk of aneuploidy in embryos increased when in vitro procedures were performed [109,110,111]. The latter-mentioned difference in the shape of the spindle upon IVM was characterized by a barrel-shaped morphology most likely caused by the excessive incorporation of MTOCs. Consequently, a reduced γ-tubulin reservoir in the ooplasma was present. Furthermore, the in vitro conditions affected the meiotic spindle localization in such a fashion that spindles in IVM oocytes were localized at a greater distance from the oolemma, whereas the spindles of in vivo matured oocytes were located much closer to the oolemma [111,112,113]. Finally, it has been shown that the murine meiotic spindle is susceptible to endocrine disruption factors, such as bisphenol B, which significantly affects the α-tubulin acetylation. This leads to spindle assembly disturbances, improper chromosome alignment, and meiotic failure [114].

The undisturbed functioning of the meiotic spindle during the first and second meiotic divisions of the oocyte is critical for producing viable oocytes and significantly determines the future development of the embryo, the successful establishment of pregnancy, and the ultimate success—healthy offspring. Abnormalities in the meiotic spindle in oocytes lead to disorders of chromosome segregation and, consequently, aneuploidy of the embryo [115], which usually leads to miscarriages or congenital defects in the offspring. By contrast, the occurrence of aneuploidy in spermatozoa is independent of paternal age, occurs only occasionally, and is mainly related to sex chromosomes [116,117]. Effective selection in favor of “chromosomally normal” pregnancies means that most aneuploid oocytes do not survive, leading to the termination of the pregnancy before its clinical confirmation. Hence, it is challenging to determine the exact rate of aneuploidy in human preimplantation embryos.

**Figure 3:**
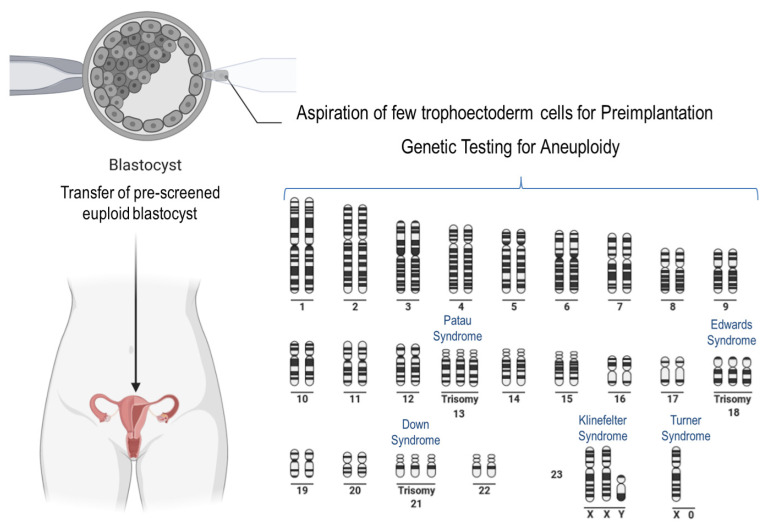
Scheme showing well-known anomalies of the human karyotype. The trisomies of chromosomes 13, 18, and 21 are shown. Moreover, two common aneuploidies of the sex-chromosomes are shown. The procedure of preimplantation genetic testing for aneuploidy prior to human embryo transfer is available only in some countries because of ethical reasons.

Well-known phenotypes associated with aneuploidy in humans are trisomies of chromosome 21 (Down syndrome), chromosome 18 (Edwards syndrome), and chromosome 13 (Patau syndrome) (Figure 3). In the latter two, affected newborns usually do not survive more than a few months. Trisomies of other autosomal chromosomes result in more severe defects in the fetuses, which rarely survive to birth. Notably, in the case of embryonic anomalies that are incompatible with life, implantation failure and/or miscarriage at different stages of pregnancy occur very often, manifesting a very common impact on female reproduction at different maternal ages [118]. For example, trisomy of chromosome 16 is often associated with spontaneous abortion [119]. Aneuploidies associated with sex chromosomes are less harmful. The most common is Klinefelter syndrome (XXY) in males, characterized by the presence of an extra X chromosome [120]. Turner syndrome (X0) in females is the only viable monosomy, whereas autosomal monosomies are lethal in humans [121]. 

Unfortunately, there are limited options to select euploid human oocytes for in vitro fertilization under clinical conditions, as techniques that allow the detailed visualization of the spindle cannot be performed because of their invasive nature. However, the use of polarized light microscopy (commonly available in IVF laboratories) is not satisfactory, as it provides insufficient information on spindle morphology. Another type of aneuploidy is a consequence of mitotic errors that occur during early embryonic cleavages. In some cases, aneuploid embryos contain both euploid and aneuploid blastomeres, but these blastocysts are not selected for embryo transfer [122]. Furthermore, mosaicism is observed not only in morphologically poor embryos but also in those with correct morphology that develop into the highest quality blastocysts [123,124]. Recent observations in human embryos revealed that mosaic embryos can develop a normal karyotype, which suggests that there is an efficient natural pathway of aneuploidy elimination [125]. In this pathway, two mechanisms might be crucial: (1) preferential proliferation of euploid cells and (2) apoptotic depletion of aneuploid cells from the inner cell mass of blastocysts [126,127]. Recently, some noninvasive attempts, such as the use of spent culture media to determine the chromosomal status of embryos, have been tested. For example, Raman spectroscopy was used to detect metabolic characteristics of aneuploid embryos in the culture media [128]. The other approach assumes that the culture media or blastocoel fluid can serve as DNA sources for preimplantation diagnostics [129]. Unfortunately, none of these approaches has yet provided satisfactory results for clinical application.

Several studies provide evidence that it is worth performing preimplantation genetic testing to detect aneuploidy in embryos generated from women of advanced age, and using this procedure, the live birth rate can be increased [130,131,132,133,134]. By contrast, two other studies reported that the latter-mentioned testing method did not positively influence the live birth rate in pregnant women below the age of 35 years [135,136]. A very recent study has shown that the percentage of live-born offspring following a conventional IVF procedure could be increased to 81.8% through the genetic testing of the blastocyst compared with that of untested embryos, in which the rate was only 77.2% [137]. It must be acknowledged that in this study, mosaic embryos were excluded from transfer for safety reasons. Considering that mosaicism is not present at the zygote stage and that the majority (about 90%) of human aneuploidies are of maternal origin [138], it seems reasonable to opt for polar body diagnosis (PBD). On the other hand, there is only a short time window of about 20 h (between sperm penetration and pronuclei appearance) to perform PBD [139]. However, this is the only way to check the ploidy status of the embryo (albeit indirectly) in countries where a preimplantation genetic diagnosis of the embryo is not allowed because of legislation. The incidence of embryo mosaicism, as assessed by trophectoderm biopsy, is estimated to be 3 to 20% [140]. Several studies have shown that mosaic embryos may develop into viable euploid newborns, with a live birth rate varying from 30 to 47% [141,142,143].

On the basis of the above-mentioned studies, one can conclude that the generation of aneuploid gametes due to factors such as aging and the environment can have fatal consequences and, therefore, a significant clinical relevance for female patients. Especially in oocytes, the process of spindle formation and the involved proteins and pathways are very sensitive to the earlier mentioned factors, which can give rise to aneuploid embryos. Reasonable testing for aneuploidy should be performed if possible.

## 5. Conclusions

In conclusion, the proper arrangement of the meiotic spindle is essential for the euploidy of human oocytes. The challenging mechanisms, particularly of meiosis I spindle formation, are, however, progressively decompensated in individuals of advanced maternal age. A growing number of studies have shown that the process of spindle formation in oocytes is a critical determinant of oocyte developmental ability and the survival of the early embryo. This has sparked the interest of researchers studying molecular reproductive medicine to better understand, diagnose, and potentially prevent age-related spindle defects and meiotic nondisjunction. Advances and improvements in assisted reproductive technologies and biotechnologies over the past thirty years have made it possible to create animal models to study the interplay of individual pathways and processes in oocytes during meiosis. All in all, our review aimed to stimulate new research ideas on this interesting topic that could help in the development of new animal models.
